# Draft Genome Sequence of the Mucoid *Pseudomonas aeruginosa* Clinical Isolate PA34

**DOI:** 10.1128/genomeA.01307-17

**Published:** 2017-11-16

**Authors:** Lucas B. Harrison, Nancy D. Hanson

**Affiliations:** Department of Medical Microbiology and Immunology, Creighton University, Omaha, Nebraska, USA

## Abstract

*Pseudomonas aeruginosa* is a serious threat to patients suffering from cystic fibrosis. These organisms are exposed to a unique set of selective pressures within the lung. Here, we report the draft genome sequence of a mucoid *P. aeruginosa* clinical isolate obtained from a cystic fibrosis patient colonized with *P. aeruginosa*.

## GENOME ANNOUNCEMENT

Chronic *Pseudomonas aeruginosa* lung infections increase the morbidity and mortality of patients suffering from cystic fibrosis (1). These chronic infections can be caused by hypermutable *P. aeruginosa* populations colonized in the lung, leading to the emergence of an antibiotic-resistant phenotype (2, 3). This genome announcement details the sequence of PA34, a mucoid strain of *P. aeruginosa* isolated from the sputum of a 17-year-old patient suffering from cystic fibrosis with recurrent *P. aeruginosa* infections. This clinical isolate was resistant to piperacillin, gentamicin, amikacin, and ceftazidime while showing an intermediate resistance pattern to both tobramycin and cefepime (4).

DNA was harvested from cultures grown overnight at 37°C in 10 ml of Mueller-Hinton broth under agitation using a Qiagen DNeasy blood and tissue DNA extraction kit. Genomic DNA was further purified using an Amicon Ultra centrifugal filtration column. Sequencing libraries were prepared using the Illumina Nextera XT DNA sample preparation kit using version 3 chemistry and optimized for 300-bp paired-end reads (5).

The PA34 genome assembly was performed using both de Bruijn and string graph assemblers, and the resulting contigs were initially positioned using reference sequences of eight *Pseudomonas aeruginosa* isolates (GenBank accession no. CP007224, AP014839, CP012066, CP002496, CP007147, NC_011770, CP014948, and CP007399). Specifically, an initial *de novo* assembly was generated using SPAdes 3.10 and improved with RAGOUT to inform contig order and AlignGraph to close the gaps between scaffolds (6–8). The assembly was improved through iterative remapping of the reads to the assembly using iCORN2 to correct amplification errors and indel assembly artifacts (9). A separate library of contigs was generated using the SGA *de novo* assembler (10). String graph assemblers, such as SGA, are better suited to assemble contigs with low coverage and highly repetitive regions, regions that are challenging to de Bruijn graph assemblers like SPAdes (10). The final assembly was performed with the SPAdes assembler using the SGA-generated contigs to supplement de Bruijn graph construction, while the initial RAGOUT- and AlignGraph- improved assembly was used for gap closure and repeat resolution (6). Contigs with a coverage less than 5× were filtered out of the assembly.

The resulting 6,302,340-bp genome had a mean read coverage of 75.59×, with 66.26% GC content. The assembly resulted in 17 contigs with *N*_50_ and *N*_75_ values of 1,682,555 and 1,069,832, respectively, and *L*_50_ and *L*_75_ values of 2 and 4, respectively. This assembly contained 6,023 genes encoding 5,863 protein-coding sequences, 65 tRNAs, 4 noncoding RNAs (ncRNAs), 13 rRNAs, and 78 pseudogenes, as identified by the NCBI Prokaryotic Genome Annotation Pipeline. ResFinder analysis of the genome identified the following antibiotic resistance genes: *bla*_OXA-50_, *bla*_PAO_, *aph*(*3′*)*-IIb*, *catB7*, and *fosA* (11).

### Accession number(s).

This whole-genome shotgun project has been deposited at DDBJ/ENA/GenBank under the accession number PDLR00000000. The version described in this paper is version PDLR01000000.
